# Massive computational identification of somatic variants in exonic splicing enhancers using The Cancer Genome Atlas

**DOI:** 10.1002/cam4.2619

**Published:** 2019-10-21

**Authors:** Kousuke Tanimoto, Tomoki Muramatsu, Johji Inazawa

**Affiliations:** ^1^ Genome Laboratory Medical Research Institute Tokyo Medical and Dental University (TMDU) Tokyo Japan; ^2^ Genomics Research Support Unit, Research Core Tokyo Medical and Dental University (TMDU), Japan Tokyo Japan; ^3^ Department of Molecular Cytogenetics Medical Research Institute Tokyo Medical and Dental University (TMDU) Tokyo Japan; ^4^ Bioresource Research Center Tokyo Medical and Dental University (TMDU) Tokyo Japan

**Keywords:** exonic splicing enhancer, nonsense‐mediated decay, somatic variants, splicing variants, TCGA

## Abstract

Owing to the development of next‐generation sequencing (NGS) technologies, a large number of somatic variants have been identified in various types of cancer. However, the functional significance of most somatic variants remains unknown. Somatic variants that occur in exonic splicing enhancer (ESE) regions are thought to prevent serine and arginine‐rich (SR) proteins from binding to ESE sequence motifs, which leads to exon skipping. We computationally identified somatic variants in ESEs by compiling numerous open‐access datasets from The Cancer Genome Atlas (TCGA). Using somatic variants and RNA‐seq data from 9635 patients across 32 TCGA projects, we identified 646 ESE‐disrupting variants. The false positive rate of our method, estimated using a permutation test, was approximately 1%. Of these ESE‐disrupting variants, approximately 71% were located in the binding motifs of four classical SR proteins. ESE‐disrupting variants occurred in proportion to the number of somatic variants, but not necessarily in the specific genes associated with the biological processes of cancer. Existing bioinformatics tools could not predict the pathogenicity of ESE‐disrupting variants identified in this study, although these variants could cause exon skipping. We demonstrated that ESE‐disrupting nonsense variants tended to escape nonsense‐mediated decay surveillance. Using integrated analyses of open access data, we could specifically identify ESE‐disrupting variants. We have generated a powerful tool, which can handle datasets without normal samples or raw data, and thus contribute to reducing variants of uncertain significance because our statistical approach only uses the exon‐junction read counts from the tumor samples.

## Graphical abstract

In this study, we computationally identified 646 somatic variants disrupting exonic splicing enhancers by compiling open access datasets from 9635 patients across 32 The Cancer Genome Atlas (TCGA) projects. The false positive rate of our method, estimated using a permutation test, was approximately 1%. Existing bioinformatics tools could not predict the pathogenicity of these variants, although these variants could cause exon skipping.

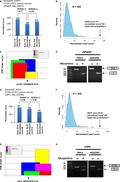

## 1 INTRODUCTION

Owing to the rapid progress of next‐generation sequencing (NGS) technologies, an enormous amount of omics data, across every type of cancer, has been analyzed and shared through public databases. These omics data, including somatic variants, whole transcriptome data, and DNA methylation profiles, have been associated with clinical information and utilized to classify cancer types based on omics profiles and explore molecular targets for therapeutics. However, in terms of their function, only a few somatic variants identified by NGS technologies have been studied, mainly because there are too many somatic variants, making functional analyses impractical. Somatic variants whose relevance to pathogenicity have not been elucidated, are called variants of uncertain significance (VUS) and are one of the problems addressed by precision medicine.^1^, ^2^, ^3^

One of the important types of somatic variants in cancer is the nonsynonymous variant, which leads to a change in the encoded amino acid. Additionally, it is known that somatic variants located in promoter regions and splice sites, which are flanking regions of exon‐intron junctions, affect gene expression and protein function.^4^, ^5^ Variants located in other cis‐regulatory elements, such as splicing enhancer and splicing silencer, also play an important role in cancer.^6^, ^7^, ^8^ Exonic splicing enhancers (ESEs) are a class of such cis‐regulatory elements. ESEs are sequence motifs located in exons and bound by SR (Serine and Arginine‐rich) proteins, which lead to the incorporation of exons into mRNA. Somatic variants that ESEs are thought to prevent binding of SR proteins to ESE sequence motifs and subsequently lead to exon skipping.^9^ In this study, we named these variants ESE‐disrupting variants. Many studies involving computational identification of ESEs using public datasets have been reported.^10^, ^11^, ^12^, ^13^, ^14^, ^15^, ^16^ For example, Woolfe et al computationally identified a number of exonic variants causing exon skipping, by utilizing datasets of ESEs and ESSs (Exonic Splicing Silencers) such as NI‐ESE, RESCUE‐ESE and so on.^11^ In another study, Mort et al predicted that exonic variants disrupt splicing, using a machine learning approach.^12^ These studies integrated analyses of genome and transcriptome datasets from different individuals. However, SR protein binding is determined not only by the genome sequence but also by epigenetics such as histone modifications,^17^ and thus, functional ESEs may differ between individual patients. Therefore, the effect of ESE on splicing is still not fully understood.

Recently, due to the establishment of international consortia to catalogue omics data from clinical samples, we can obtain paired genome‐transcriptome datasets from the same individual. Transcriptome information is important to elucidate splicing regulation. Given these facts, we hypothesized that ESE‐disrupting variants could be identified massively by compiling somatic variant and gene expression data obtained from the public database The Cancer Genome Atlas (TCGA). TCGA contains omics data and clinical information from over 12 000 patients across every type of cancer. TCGA data are classified into two types, controlled and open. Access to controlled data, including raw sequence data such as binary alignment map (BAM) format, requires user authorization and authentication. In contrast, we can easily access open data, which includes somatic variants, gene expression, DNA methylation, clinical information and so on. Here, we computationally identified somatic variants in ESEs using a variety of population genomics approaches and numerous open access datasets from TCGA.

## 2 MATERIALS AND METHODS

### 2.1 Data download

**Figure 1:**
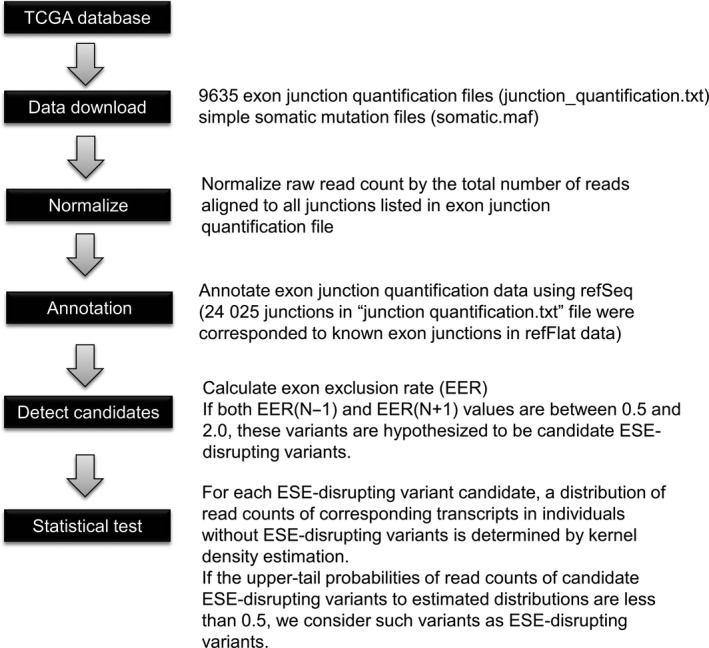
The data analysis workflow for this study

The data analysis workflow for this study is shown in Figure 1. We obtained two data type files, “Gene expression quantification” files (junction_quantification.txt) and “Simple somatic mutation” files (somatic.maf), of 9635 patients across 32 TCGA projects (25 tissues) from the “Legacy Archive” of GDC Applications. The TCGA projects used in this study are listed in Table S1. The genomic coordinates of these data are hg19. The junction_quantification.txt files contain read counts of 249 547 paired genomic coordinates. Each read count indicates the number of reads aligned to a reference genome across the gap between each paired genomic coordinate. Of these paired genomic coordinates, 24 025 genomic coordinates correspond to known exon‐exon junctions of RefSeq transcripts, which are associated with 8079 genes (analyzable genes are listed in Table S2). To compare samples, each read count was normalized by the total read counts aligned to all junctions listed in the junction_quantification.txt file of each sample.

### 2.2 Calculation of the exon exclusion rate

**Figure 2:**
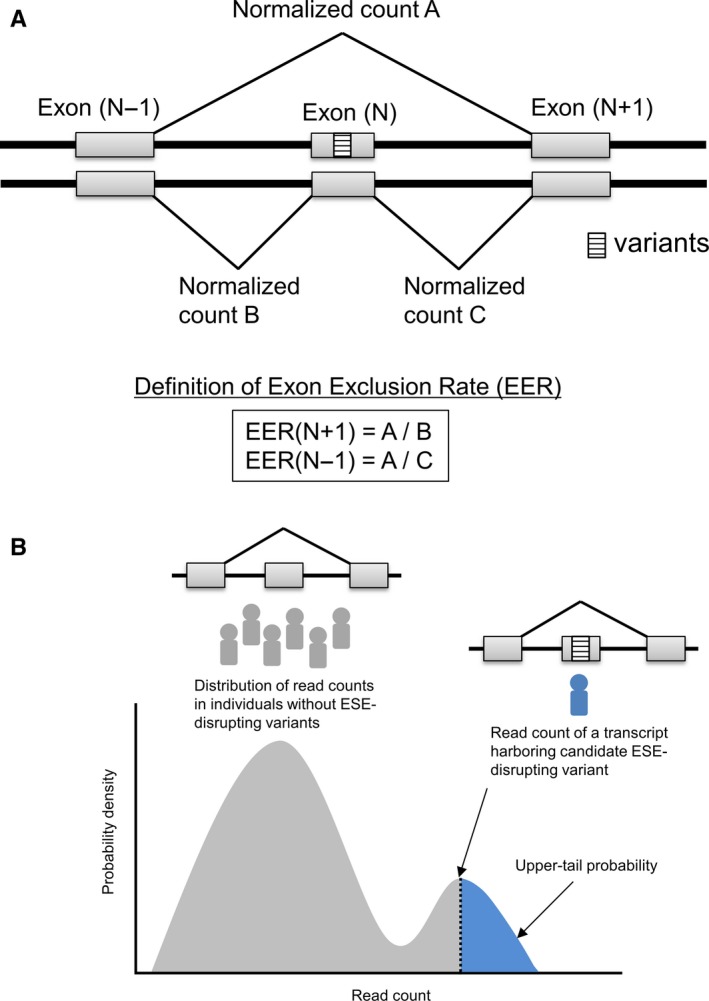
Definition of exon exclusion rate (EER) (A) and upper‐tail probability (B)

To identify ESE‐disrupting variants, we defined the exon exclusion rate (EER) (Figure 2A). EER indicates the ratio of transcripts with skipped exons containing somatic variants (which we named “Normalized count A”) to normal transcripts. To calculate EER(N − 1), read counts aligned to a junction between an exon with somatic variants and the previous exon (which we named “Normalized count B”) were used. Similarly, to calculate EER(N + 1), read counts aligned to a junction between an exon with somatic variants and the next exon (which we named “Normalized count C”) were used. If ESE disruption is caused by somatic variants, exons derived from one allele harboring somatic variants should be skipped, and the EER value in this situation is assumed to be approximately 1. Therefore, if both EER(N − 1) and EER(N + 1) values are between 0.5 and 2.0, these variants are hypothesized to be candidate ESE‐disrupting variants. Since the fraction of tumor cells is not 100%, true EER value should be below 1. However, to avoid false negatives, we used low stringency criteria at this step and performed further validation in the next step (explained below).

In this study, we focused only on autosomal variants because we could not compare RNA‐seq read counts of sex chromosomes genes between males and females. Splice site (a donor site and an acceptor site of intron) variants were not included in this study because these variants obviously regulate splicing.

### 2.3 Validation of candidate ESE‐disrupting variants

For each ESE‐disrupting variant candidate, a distribution of read counts of corresponding transcripts in individuals without ESE‐disrupting variants was determined by kernel density estimation, which is a nonparametric way to estimate the probability density function, using the generic function “density” in the R statistical software (version 3.3.0). Subsequently, if the upper‐tail probabilities of “Normalized count A” of candidate ESE‐disrupting variants to estimated distributions were less than 0.05, we considered such variants as ESE‐disrupting variants (Figure 2B). If a random variable X is given and its distribution admits a probability density function f, the upper‐tail probability of X can be calculated as $P\left(x>X\right)=1-\int_{0}^{X}f\left(x\right)dx$. In this step, a random variable is equivalent to the read count of a transcript skipping an exon harboring ESE‐disrupting variant candidate, and the probability density function is equivalent to a distribution determined by kernel density estimation.

### 2.4 Permutation test

To evaluate the false positive ratio, we repeated the procedures for identifying ESE‐disrupting variants 1000 times by permuting the combination of somatic variants and RNA‐seq data. Permutation was performed by exchanging the sample labels of the data randomly when combining somatic variants and RNA‐seq data. The combinations were permutated using in‐house Perl scripts, and other procedures were performed as described above.

### 2.5 Evaluation of the effects of nonsense variants on gene expression

We obtained rsem.genes.normalized.results.txt files of each sample across 32 TCGA projects. This file format contains normalized counts of 20 502 transcripts from RefSeq, KIAA, and FLJ. First, the distribution of expression of each gene was determined by kernel density estimation, using genes not harboring nonsense variants in each TCGA project. Next, the lower‐tail probabilities of each gene harboring nonsense variants to the distributions estimated above were calculated. The lower‐tail probability calculations were performed using the R statistical software (version 3.3.0). If a random variable *X* is given and its distribution admits a probability density function *f*, the lower‐tail probability of *X* can be calculated as $P\left(X>x\right)=1-\int_{0}^{X}f\left(x\right)dx$. At this step, a random variable is equivalent to the read count of each gene harboring nonsense variant in each sample, and the probability density function is equivalent to a distribution determined by kernel density estimation.

### 2.6 Searching for SR protein binding motifs using ESE finder

In the ESE finder release 3.0 (http://krainer01.cshl.edu/cgi-bin/tools/ESE3/esefinder.cgi?process=home) analysis, we used the default threshold. ESE finder identifies binding motifs of four SR proteins (SRSF1, SRSF2, SRSF5, and SRSF6) based on functional systematic evolution of ligands by exponential enrichment (SELEX).^18^, ^19^ ESE finder provides two SRSF1 scores, SF2/ASF and IgM‐BRCA1. In this study, when either of the two scores was above the threshold, we assumed that the input sequences contained SRSF1 binding motifs.

### 2.7 GO enrichment analysis

GO terms were obtained from the GO consortium.^20^, ^21^ The *P*‐value was calculated using a hypergeometric distribution in R statistical software.

### 2.8 Data processing

All text data used in this study was processed by in‐house Perl scripts. The scripts used in this study are available on GitHub repository under the following address:

https://github.com/ktresearch/ese_disrupting_variants

### 2.9 Cell culture and PCR‐based splicing pattern analysis using morpholino oligos

HeLa and HEK293 cell lines were purchased from the American Type Culture Collection (ATCC, Manassas, VA). All cells were grown in DMEM supplemented with 10% fetal bovine serum in a humidified atmosphere with 5% CO_2_ at 37°C and were authenticated by monitoring cell morphology.

HeLa and HEK293 cell were treated with 10 µmol/L morpholino oligos for 48 hours using Endo‐Porter (Gene Tools, LLC). Morpholino oligos were obtained from Gene Tools and were designed not to target splice sites to prevent exon skipping caused by splice site inhibition. Morpholino oligo sequences were as follows: APMAP 5′‐CAGAGCTGCTGGGCCGGATGTTGTC‐3′, USP4 5′‐ATTCAGTTGTTCTTCGCATATGCA‐3′, and DPH5 5′‐GTTCTTCTCCTCGTATTCTTTGATT‐3′. DPH5‐targeting morpholino oligo was used as a control. Total mRNA was extracted from cell lines treated with morpholino oligos, and cDNA was synthesized using the PrimeScript™ II 1st‐strand cDNA Synthesis Kit (TAKARA) according to manufacturer's instructions. PCR amplification was performed using PrimeSTAR MAX DNA polymerase (TAKARA) using the following reaction conditions: 98°C for 2 minutes, 30 cycles of 98°C for 10 seconds, 58°C for 5 seconds, 72°C for 15 seconds, and 72°C for 2 minutes. PCR primer sequences used to amplify APMAP exon 7‐9 were 5′‐GTGAAACTGCTGCTGTCCTC‐3′ (Fw) and 5′‐GGCACAAACTTCATCACCGT‐3′ (Rv). PCR primer sequences used to amplify USP4 exon 20‐22 were 5′‐ACCTGTCAGCAAGGCCTTAT‐3′ (Fw) and 5′‐AGGATCGTGGAGTCAGCATT‐3′ (Rv).

## 3 RESULTS

### 3.1 Identification of ESE‐disrupting variants

We attempted to computationally identify somatic variants located in ESEs, which perturb their function (named ESE‐disrupting variants), by the integrated analysis of gene expression data and somatic variants from TCGA. The data analysis workflow is shown in Figure 1. To identify ESE‐disrupting variants, we defined the EER as shown in Figure 2. EER indicates the ratio of transcripts with skipped exons to normal transcripts. We calculated the EER of 156 794 somatic variants obtained from TCGA across 32 TCGA projects and selected candidate ESE‐disrupting variants by the procedure described in Section 2.

To assess whether exon skipping is caused by ESE‐disrupting variants, tumor and corresponding normal tissue RNA‐seq data are needed. However, TCGA contains fewer normal tissue data compared to tumor data. Thus, to validate whether exon skipping in these candidates was statistically significant, we calculated an upper‐tail probability for each candidate. This compares the degree of exon skipping in samples with somatic variants to that in corresponding control samples without somatic variants (see Section 2). Finally, we obtained 646 ESE‐disrupting variants by this validation step. Details of the 646 ESE‐disrupting variants obtained are shown in Table S3.

### 3.2 Permutation test

**Figure 3:**
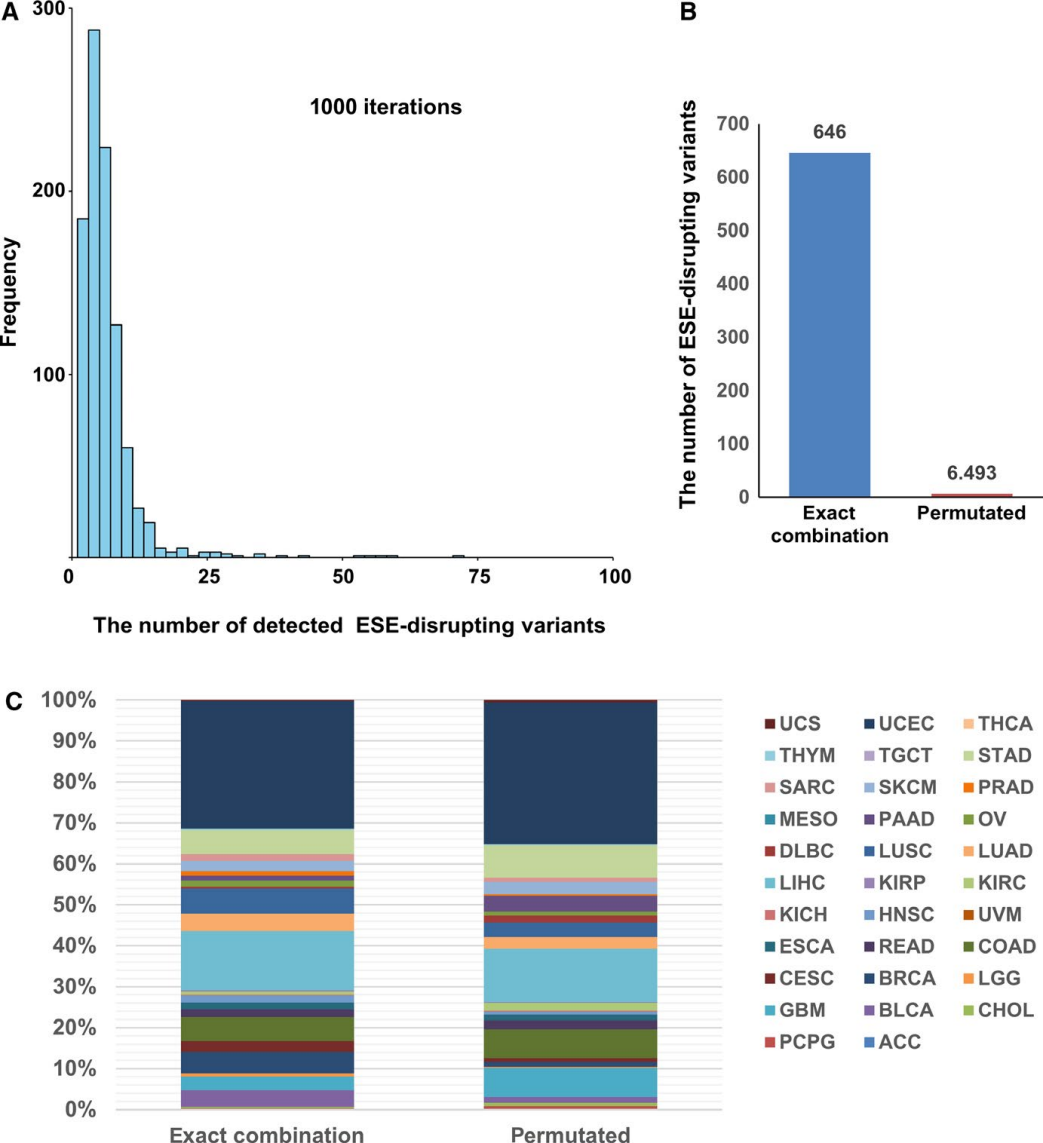
Permutation test. A, Distribution of number of ESE‐disrupting variants obtained after the permutation test. B, Numbers of ESE‐disrupting variants detected in exact combination and permutated combinations. The number of permutated combinations is an average of 1000 combinations. C, Details of ESE‐disrupting variants detected in each TCGA project

To estimate the number of false positive ESE‐disrupting variants, we performed a permutation test. A permutation test is one of the standard approaches to determine statistical significance in genome‐wide association studies (GWAS) and other integrative analyses.^22^, ^23^, ^24^, ^25^ Permuting the combination of genetic information and traits randomly and repeating the analysis, provides a null distribution while maintaining the correlation structure of the datasets.^26^ To evaluate the false positive rate, we repeated the procedures for identifying ESE‐disrupting variants 1000 times by randomly permuting the combinations of somatic variants and RNA‐seq data. The distribution of the number of ESE‐disrupting variants obtained from this test is shown in Figure 3A. The average and median false positive ESE‐disrupting variants were 6.493 and 5 respectively. The average number of false positives was approximately 1% of the ESE‐disrupting variants using exact combination (Figure 3B). The ratio between exact and permutated combinations was similar in each TCGA project (Figure 3C). This test indicates that very few false positives were detected by our method.

### 3.3 Summary of ESE‐disrupting variants identified

**Figure 4:**
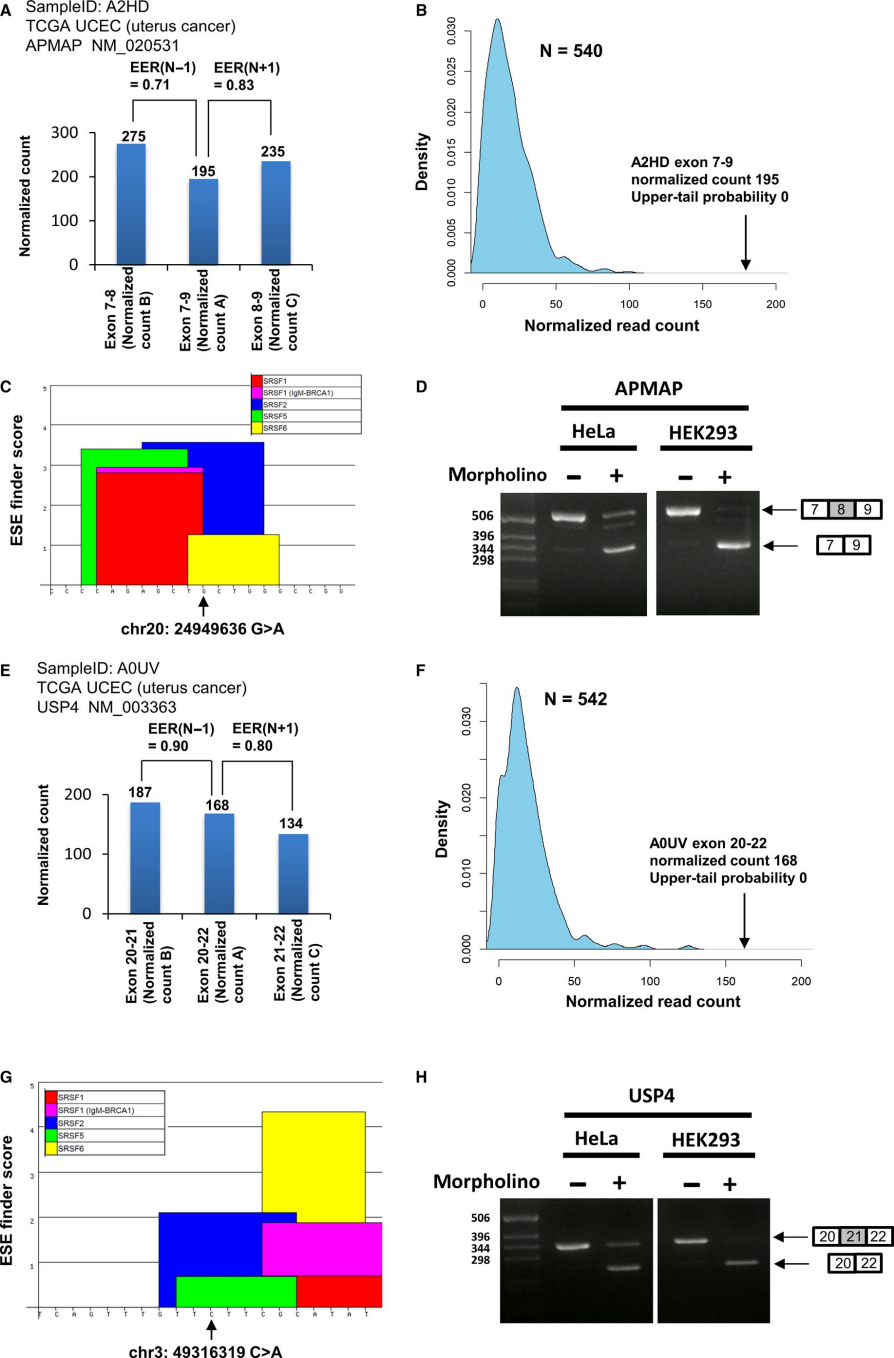
Examples of ESE‐disrupting variants identified. A, Normalized read counts of each exon‐exon junction around the APMAP gene somatic variant in the uterus cancer A2HD sample. B, Estimated normalized count distribution of exons 7‐9 without variants in APMAP in TCGA UCEC project samples (N = 540) determined by kernel density estimation. C, ESE finder graphical result of exon 8 somatic variant (chr20: 24949636 G>A) in the APMAP gene from sample A2HD. D, PCR‐based splicing pattern analysis by morpholino oligos targeting an ESE‐disrupting variant in exon 8 of the APMAP gene in HeLa and HEK293 cell lines. E, Normalized read counts of each exon‐exon junction around the USP4 gene somatic variant in the uterus cancer A0UV sample. F, Estimated normalized count distribution of exons 20‐22 without variants in USP4 in TCGA UCEC project samples (N = 542) determined by kernel density estimation. G, ESE finder graphical result of exon 21 somatic variant (chr3: 49316319 C>A) in the USP4 gene from sample A0UV. H, PCR‐based splicing pattern analysis by morpholino oligos targeting an ESE‐disrupting variant in exon 21 of the USP4 gene in HeLa and HEK293 cell lines

The summary of ESE‐disrupting variants identified by our method is shown in Table 1. Of the 156 794 somatic variants analyzed in this study, 0.41% (0%‐0.72%) were identified as ESE‐disrupting variants. The types of cancer not harboring ESE‐disrupting variants tended to have fewer samples. Examples of ESE‐disrupting variants identified are shown in Figure 4. A TCGA UCEC project (uterus cancer) sample A2HD harbored a G > A silent (synonymous) variant (chr20:24949636) in exon 8 of the APMAP gene. The values of EER(N + 1) and EER(N‐1) for this variant were 0.83 and 0.71 respectively (Figure 4A). In TCGA UCEC project, 540 samples harbored no somatic variants in exon 8 of the APMAP gene, and the read count distribution of their exons, determined by kernel density estimation, is shown in Figure 4B. To this distribution, the upper‐tail probability of the read count, skipping exon 8 in sample A2HD, was 0. We searched for SR protein binding motifs around this somatic variant, using all available SR proteins (SRSF1, SRSF2, SRSF5, SRSF6) in the ESE finder, and found that this variant probably disrupted SRSF2 and SRSF6 binding motifs (Figure 4C). Furthermore, we experimentally validated that this variant was located in an ESE, using HeLa and HEK293 cells. We used morpholino oligos to block the region, corresponding to the region containing the ESE‐disrupting variants, in both cell lines and found that this led to exon skipping (Figure 4D). Our morpholino oligos specifically blocked the target region (Figure S1).

**Table 1:** The numbers of somatic variants analyzed in this study and the ESE‐disrupting variants identified

TCGA project	The number of samples	The number of total variants	The number of ESE‐disrupting variants	The percentage of ESE‐disrupting variants
ACC	79	538	0	0
PCPG	179	336	2	0.60
CHOL	36	308	2	0.65
BLCA	408	5084	27	0.53
GBM	154	4600	21	0.46
LGG	516	2228	5	0.22
BRCA	1092	9079	34	0.37
CESC	305	3742	17	0.45
COAD	431	11 507	38	0.33
READ	151	2555	12	0.47
ESCA	182	2638	11	0.42
UVM	80	158	0	0
HNSC	520	4068	11	0.27
KICH	66	258	1	0.39
KIRC	531	1227	5	0.41
KIRP	290	1202	2	0.17
LIHC	371	38 398	94	0.24
LUAD	516	7204	27	0.37
LUSC	500	9198	40	0.43
DLBC	48	773	3	0.39
OV	304	1822	9	0.49
PAAD	177	2696	8	0.30
MESO	87	175	0	0
PRAD	497	1451	7	0.48
SKCM	104	2229	16	0.72
SARC	258	1854	11	0.59
STAD	379	7299	38	0.52
TGCT	147	344	0	0
THYM	120	1048	2	0.19
THCA	505	494	0	0
UCEC	545	31 875	201	0.63
UCS	57	406	2	0.49
Total	9635	156 794	646	0.41

Another example is shown in Figure 4E‐H. A TCGA UCEC project (uterus cancer) sample, A0UV, harbored a C > A missense variant (chr3:49316319) in exon 21 of the USP4 gene. The values of EER(N + 1) and EER(N − 1) for this variant were 0.90 and 0.80 respectively (Figure 4E). In TCGA UCEC project, 542 samples harbored no somatic variants in exon 21 of the USP4 gene, and the read count distribution of their exons, determined by kernel density estimation, is shown in Figure 4F. The upper‐tail probability of the read count skipping exon 21 in sample A0UV was 0. Using the ESE finder, we estimated that this variant probably disrupted SRSF2 and SRSF5 binding motifs (Figure 4G). We validated that this variant was located in an ESE in both cell lines by morpholino experiments (Figure 4H).

### 3.4 Characteristics of ESE‐disrupting variants

**Figure 5:**
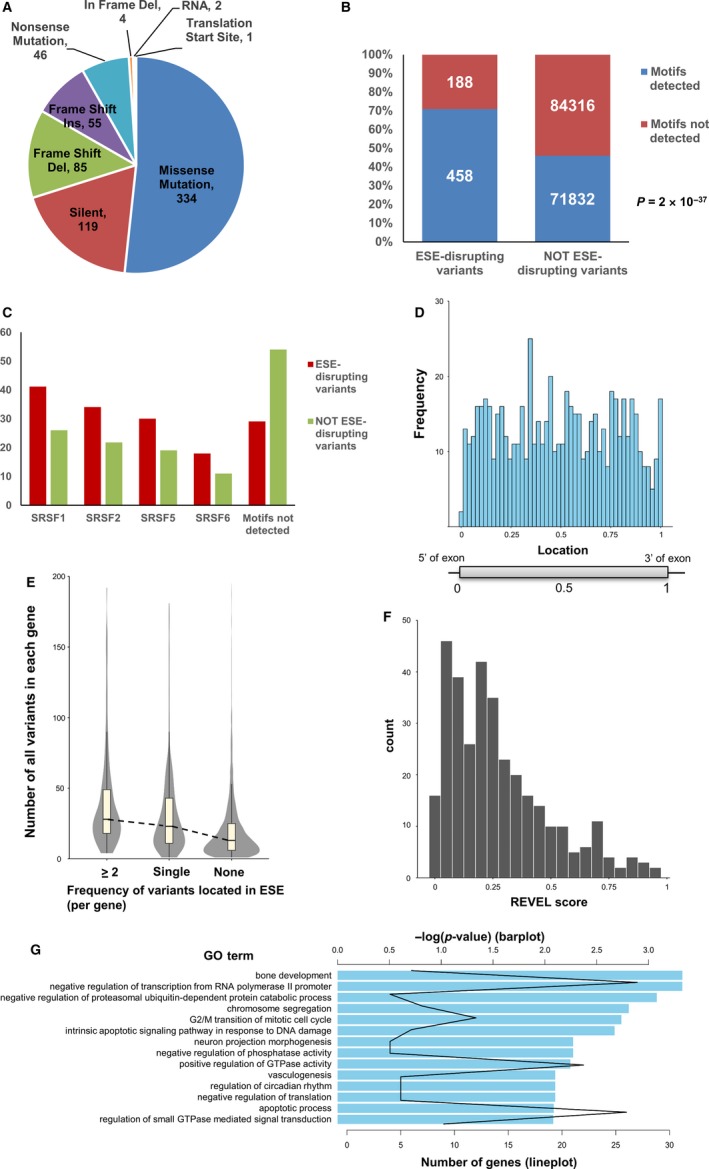
Characteristics of identified ESE‐disrupting variants. A, Details of variant types of ESE‐disrupting variants identified. B, Numbers of somatic variants located in four known SR protein (SRSF1, SRSF2, SRSF5, SRSF6) binding motifs, identified by ESE finder. *P*‐value was calculated using a hypergeometric distribution. C, Percentages of variants located in SR protein binding motifs identified by ESE finder. D, Positional distribution of identified ESE‐disrupting variants in exons. The X‐axis indicates the normalized position of each exon; 0 indicates the 5ʹ end of the exon and 1 indicates the 3ʹ end of the exon. E, Distributions of the number of total somatic variants in each gene. Genes were classified into three groups: more than two ESE‐disrupting variants detected, one ESE‐disrupting variant detected, and no ESE‐disrupting variants detected. F, REVEL score distribution of identified ESE‐disrupting variants. The X‐axis indicates the REVEL score. G, Results of GO enrichment analysis using genes harboring ESE‐disrupting variants. The bar plot indicates the log_10_ converted *P*‐value for each GO term. The line plot indicates the number of genes associated with each GO term

Details of variant types of ESE‐disrupting variants identified are shown in Figure 5A. Of these, 18% were synonymous variants. We analyzed whether the ESE‐disrupting variants identified in this study were located in known binding motifs of four SR proteins using the ESE finder. We found that approximately 71% of the variants were located in the binding motifs of the four SR proteins (Figure 5B), and the fraction associated with each SR protein was similar (Figure 5C). On the other hand, of the 156 148 somatic variants identified as non‐ESE‐disrupting variants, approximately 46% were located in the binding motifs of the four SR proteins (Figure 5B). The binding motifs of the four SR proteins were significantly enriched in ESE‐disrupting variants identified in this study (*P* = 2 × 10^−37^, hypergeometric distribution). Details of motifs detected by the ESE finder are shown in Table S4.

It is known that ESEs are located in various regions from the 5′ to the 3′ end of the exon.^13^ We examined the positional distribution of ESE‐disrupting variants identified in this study. To examine the positional distribution, the distance from each ESE‐disrupting variant to the 5′ end of the exon was normalized by the length of the exon. We found that the ESE‐disrupting variants were located uniformly across exons (Figure 5D).

To evaluate the correlation in the frequencies between somatic variants and ESE‐disrupting variants, the genes analyzed in this study were classified into three groups: more than two ESE‐disrupting variants detected, one ESE‐disrupting variant detected, and no ESE‐disrupting variants detected. The distribution of each group is shown in Figure 5E. The genes tended to contain ESE‐disrupting variants in proportion to the number of somatic variants.

We computed the effect of 334 missense ESE‐disrupting variants on protein function using the REVEL tool.^27^ REVEL is a tool used to quantify the pathogenicity of somatic variants by integrating 13 algorithms predicting the effect of variants on protein structure and function. Of the 334 missense variants, 188 (56%) ESE‐disrupting variants scored below 0.25, which were judged to have a low probability of causing disease (Figure 5F). This result suggests that existing bioinformatics tools regarded most ESE‐disrupting variants identified in this study as having low pathogenicity, although these variants could cause exon skipping.

To examine the correlation between the identified ESE‐disrupting variants and gene function, we performed a GO enrichment analysis. Not only cancer‐associated processes, for example, cell cycle (GO:0000086 G2/M transition of mitotic cell cycle) and apoptosis (GO:0006915 apoptotic process, GO:0008630 intrinsic apoptotic signaling pathway in response to DNA damage),^28^ but also other biological processes were enriched (Figure 5G). Furthermore, we examined whether the genes that represented oncogenic signatures were enriched in the set of genes harboring ESE‐disrupting variants identified in this study. Of 8079 analyzable genes, 4551 genes were a part of oncogenic signatures in MSigDB.^29^, ^30^, ^31^ We identified ESE‐disrupting variants in 416 genes across 32 TCGA projects. Among these, 243 genes were identified as ESE‐disrupting variants (*P* = .234, hypergeometric distribution).

### 3.5 Nonsense‐mediated decay may be avoided by exon skipping caused by ESE‐disrupting variants

**Figure 6:**
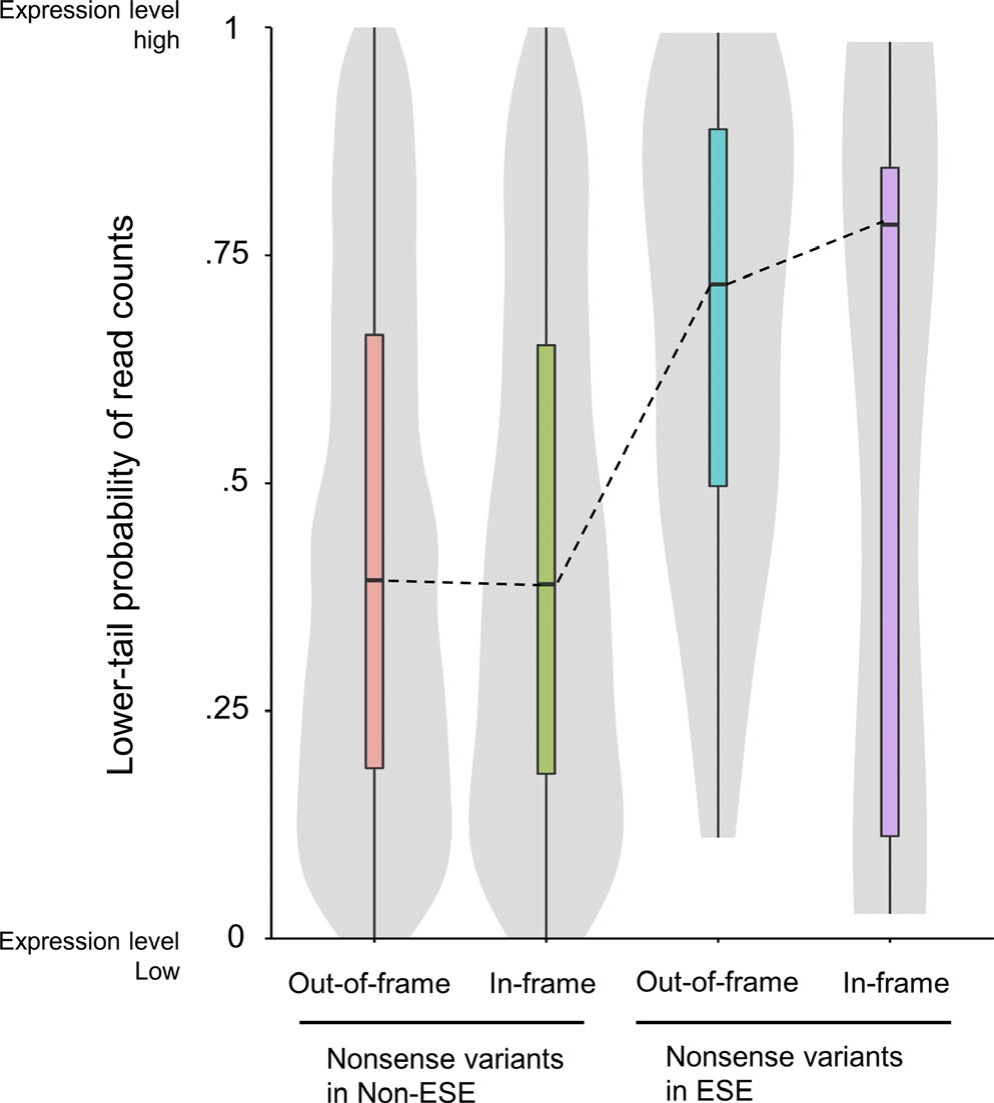
Expression levels of genes harboring nonsense variants. The *Y*‐axis shows a lower‐tail probability of read counts of genes harboring ESE‐disrupting nonsense variants to the read count distributions of samples without nonsense variants. This value can range from 0 to 1, with a larger value indicating higher expression levels. Genes were classified into four groups: out‐of‐frame variants in non‐ESE, in‐frame variants in non‐ESE, out‐of‐frame variants in ESE and in‐frame variants in ESE

Transcripts harboring nonsense variants can be potentially degraded by nonsense‐mediated decay (NMD),^32^ which is an mRNA quality control system. However, nonsense variants identified as ESE‐disrupting variants cause exon skipping and thus, do not trigger NMD.^33^ Therefore, we evaluated the effects of nonsense variants, identified as ESE‐disrupting variants, on gene expression using TCGA gene expression datasets. To evaluate this, we calculated lower‐tail probabilities of 5546 nonsense variants used in this study, including 43 ESE‐disrupting nonsense variants, by the procedure described in Section 2. A lower‐tail probability can range from 0 to 1, with a larger value indicating that samples harboring nonsense variants have higher expression levels than samples without nonsense variants. Nonsense variants were classified into four types: out‐of‐frame variants in non‐ESE, in‐frame variants in non‐ESE, out‐of‐frame variants in ESE, and in‐frame variants in ESE. The out‐of‐frame variants in ESE cause exon skipping and a subsequent frameshift. The in‐frame variants in ESE cause exon skipping but maintain the reading frame. The lower‐tail probability distributions for each group are shown in Figure 6. The medians of both groups, in which nonsense variants were not located in the ESE, were less than 0.50. This result demonstrates that the genes harboring nonsense variants, not identified as ESE‐disrupting, tended to have lower expression levels compared to those without nonsense variants. This tendency is presumably caused by NMD. On the other hand, the medians of both groups in which nonsense variants were located in the ESE were more than 0.50. This result demonstrates that the genes harboring nonsense variants, identified as ESE‐disrupting, tended to have higher expression levels than those without nonsense variants. This suggests that the transcripts harboring ESE‐disrupting nonsense variants tend to escape NMD surveillance, and thus transcripts lacking exons harboring nonsense variants accumulate. Details of the lower‐tail probabilities of each nonsense variant are shown in Table S5.

## 4 DISCUSSION

In this study, we performed an integrated analysis of somatic variants and gene expression data and identified 646 ESE‐disrupting variants across 32 TCGA projects. The false positive rate of our method was estimated to be approximately 1% by the permutation test (Figure 3). The statistical test, using the distribution of gene expression levels of the samples not harboring somatic variants in the validation step, probably reduced false positives. GO enrichment analysis showed that ESE‐disrupting variants occurred in genes associated with various biological processes (Figure 5G). It is well known that aberrant splicing in cancer frequently occurs in genes associated with cancer‐related processes, such as transcription factor, cell signaling, proliferation, invasion, and metastasis.^34^

ESE‐disrupting variants were significantly biased toward UCEC and SKCM (Figure S2A). Regarding the genomic loci, ESE‐disrupting variants were significantly biased toward chromosome 3p, 5q, 8q, 16p, and 22q (Figure S2B). These biases were found in groups that had more variants than average. However, other factors such as epigenetics may influence these biases because they were not found in some highly mutated groups such as LIHC. Further analysis is required to explain these biases. Our results suggest that ESE‐disrupting somatic variants occur in proportion to the total number of somatic variants, not in specific genes associated with carcinogenesis or cancer progression (Figure 5E,G). ESE‐disrupting variants identified in this study were located uniformly across exons (Figure 5D). Using raw sequence data across six types of cancer from TCGA, Hyunchul Jung et al demonstrated that most of the somatic variants with abnormal splicing were enriched in nucleotides flanking exon‐intron junctions, which include splice sites, while others were located uniformly in exonic regions.^35^ Our results were consistent with their study, except for variants in exon‐intron junctions. In this study, somatic variants in splice sites were not analyzed because such variants clearly regulate splicing. This may be one of the reasons why only 0.41% of total variants were identified as ESE‐disrupting by our method.

The ESE finder can identify binding motifs of four SR proteins namely, SRSF1, SRSF2, SRSF5, and SRSF6. These SR proteins are categorized as “classical” SR proteins, which have structural and functional similarities.^36^ Approximately 71% of ESE‐disrupting variants identified in this study were located in one of these four classical SR protein binding motifs (Figure 5B). If additional SR protein motifs were included, we may obtain a different result because each SR protein has multiple binding motifs and targets.^37^ However, the conclusion that our method could specifically identify ESE‐disrupting variants may not change.

Of the ESE‐disrupting variants identified in this study, 17% were synonymous variants (Figure 5A). Additionally, 56% of the ESE‐disrupting variants were predicted to have low pathogenicity by REVEL analysis (Figure 5F). Thus, the variants identified in this study may have escaped functional analyses because cancer researchers generally to focus on changes in protein structure and function. However, somatic variants causing transcriptional alterations possibly play an important role in cancer.^35^, ^38^, ^39^, ^40^ Furthermore, transcripts escaping NMD surveillance, as shown in Figure 6, would lead to an accumulation of aberrant transcripts, which lack exons harboring nonsense variants. These transcripts have an impact on protein functions, with or without an associated frameshift.^41^ Taken together, the pathogenicity of every variant including synonymous variants may not be negligible with regards to cancer. Our approach is a useful tool to detect such pathogenic somatic variants not identified by conventional methods.

Recently, several studies have reported the identification of splicing patterns using TCGA datasets. Kahles et al identified approximately 173 000 tumor‐specific alternative splicing events and 251 000 exon‐exon junctions using tumor datasets from 8705 patients.^42^ Shirley et al showed that 341 486 variants had a significant impact on mRNA splicing, and approximately 70% of these variants were not registered in the dbSNP database.^43^, ^44^ Furthermore, these studies could analyze all genes and identify novel splicing variants because of integrated analyses using raw sequence data such as FASTQ or BAM files. In studies using TCGA datasets, one of the things to be considered is how to handle data from normal samples because tumor matched normal samples are a lot fewer than only tumor samples in TCGA. For example, Kahles et al only used tumor types that had at least 50 tumor samples and 10 matched normal samples, to analyze differential splicing events between tumor and normal tissue. Shirley et al merged normal samples derived from different tissues, in order to analyze tumor types which did not have enough normal samples. On the other hand, our statistical approach, using kernel density estimation, does not need normal samples. To validate ESE‐disrupting variant candidates, we calculated the upper‐tail probability to the distribution of the gene expression levels of tumor samples not harboring corresponding variants, using kernel density estimation. Although our method could not detect ESE‐disrupting variants in genes whose expression levels were similar to those of control tumor samples, our population genomics approach can detect ESE‐disrupting variants overlooked by other methods. In fact, of the ESE‐disrupting variants identified in our study, only about 4% of variants were identified by Shirley and colleagues (Table S3).

Compared to these previous studies, we recognize that our approach is not comprehensive because junction_quantification.txt files from TCGA “Legacy Archive” used in this study contains exon‐exon junction information from a limited number of known transcripts (Table S2). This may also be correlated with a low identification percentage for all somatic variants. Whereas analysis of tumor‐normal paired raw sequence data such as fastq or BAM formats enables us to perform a comprehensive analysis, access to this type of data is restricted, owing to personal and ethical issues. However, edited datasets of tumor samples such as VCF format data, gene expression data and clinical information generated by international consortia are numerous and easily accessible. Our method provides a powerful tool to handle large datasets without normal samples or raw data. We hope that our approach will reduce VUS and contribute to cancer biology and clinical treatment.

## CONFLICTS OF INTEREST

The authors declare no conflicts of interest associated with this manuscript.

## Data Availability

The data that support the findings of this study are available in The Cancer Genome Atlas at https://portal.gdc.cancer.gov/.
