# Supplementary figures and images for: Massive computational identification of somatic variants in exonic splicing enhancers using The Cancer Genome Atlas
Source: Cancer Med. 2019 Oct 21;8(17):7372–84. doi: 10.1002/cam4.2619 (PMC6885893; doi:10.1002/cam4.2619)

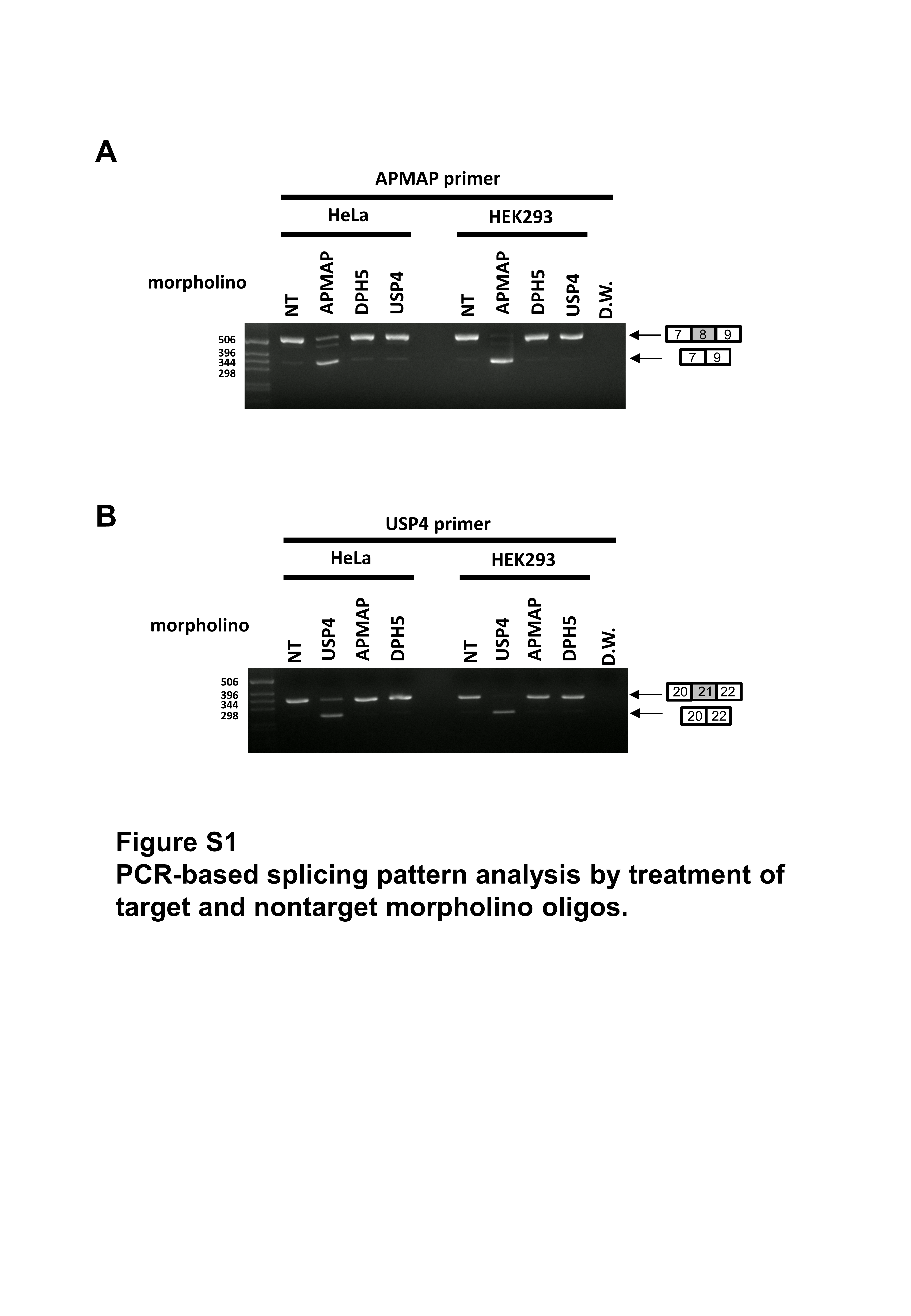

Supplement: Supplementary file 1 [file CAM4-8-7372-s001.tif]

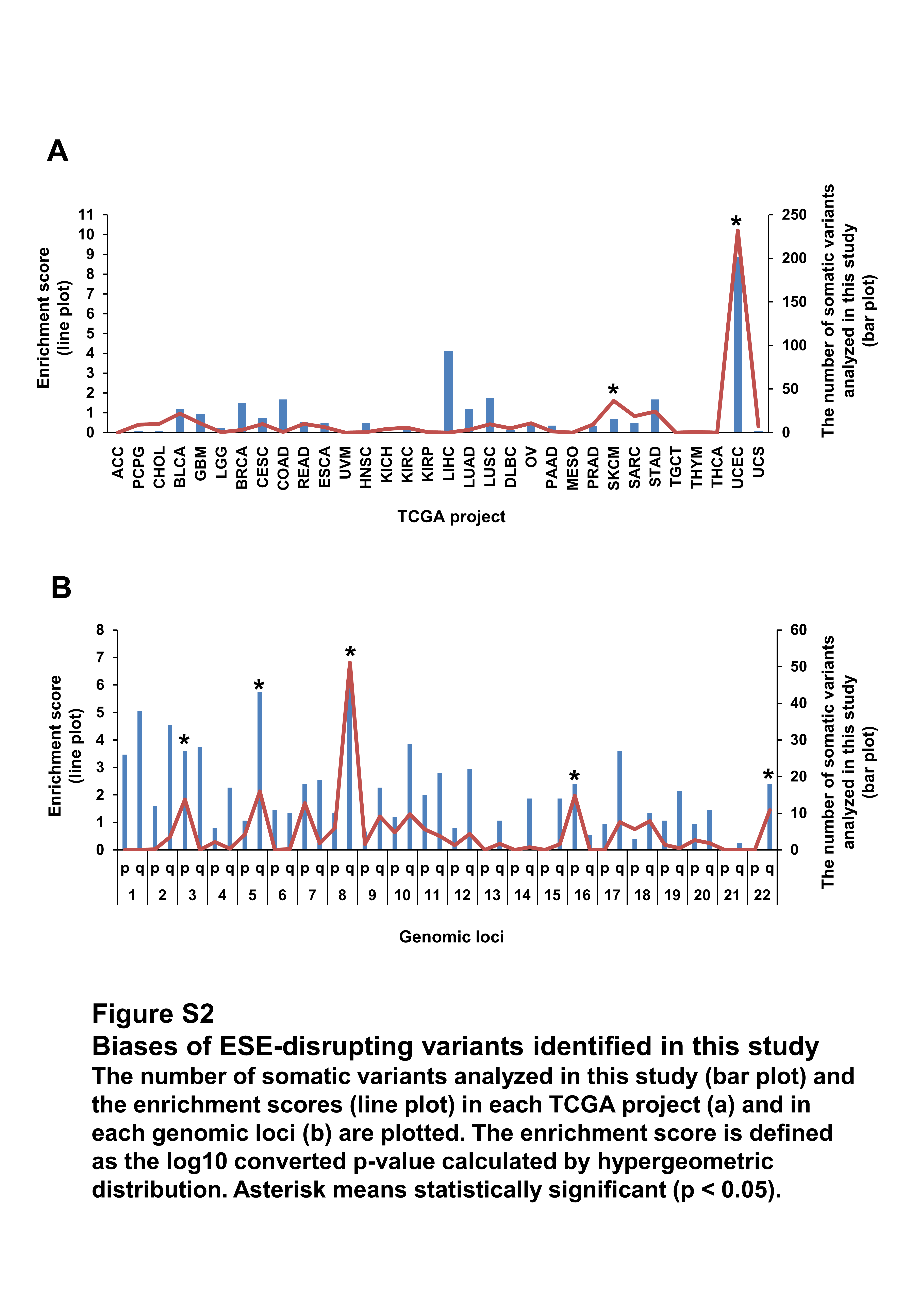

Supplement: Supplementary file 2 [file CAM4-8-7372-s002.tif]
